# Klippel-Trenaunay and Parkes-Weber syndromes: two case reports

**DOI:** 10.1590/1677-5449.005417

**Published:** 2017

**Authors:** Carlos Alberto Araujo Chagas, Lucas Alves Sarmento Pires, Marcio Antonio Babinski, Tulio Fabiano de Oliveira Leite

**Affiliations:** 1 Universidade Federal Fluminense – UFF, Department of Morphology, Niteroi, RJ, Brazil.; 2 Universidade de São Paulo – USP, School of Medicine, Unit of Interventional Radiology, São Paulo, SP, Brazil.

**Keywords:** Klippel-Trenaunay syndrome, Parkes-Weber syndrome, angiodysplasia, nevus, arteriovenous malformations, síndrome de Klippel-Trenaunay, síndrome de Parkes-Weber, angiodisplasias, nevus, malformações arteriovenosas

## Abstract

Parkes-Weber syndrome is a congenital vascular disease that comprises capillary, venous, lymphatic, and arteriovenous malformations. Although Parkes-Weber syndrome is a clinically distinct entity with serious complications, it is still frequently misdiagnosed as Klippel-Trenaunay syndrome, which consists of a triad of malformations involving the capillary, venous, and lymphatic vessels, without arteriovenous fistulas. Both syndromes are generally diagnosed with Doppler ultrasound and confirmed by magnetic resonance angiography. The aim of this study is to describe one case of Klippel-Trenaunay syndrome, in a 36-year-old patient, and one case of Parkes-Weber syndrome, in a 21-year-old patient. We review the literature in order to discuss the possible causes and consequences of these diseases related to venous hypertension and angiodysplasia, taking a clearer approach to their differences, and discussing their treatment.

## Resumo

A síndrome de Parkes-Weber é uma doença vascular congênita que consiste em malformações capilares, venosas, linfáticas e arteriovenosas. Embora seja uma entidade clinicamente distinta com complicações graves, essa síndrome ainda é frequentemente diagnosticada erroneamente como síndrome de Klippel-Trenaunay, que consiste em uma tríade de má formação nos vasos capilares, venosos e linfáticos, sem fístula arteriovenosa. Ambas as síndromes são geralmente diagnosticadas através de ultrassom Doppler e confirmadas pela angiografia por ressonância magnética. O objetivo deste estudo é descrever um caso de síndrome de Klippel-Trenaunay em um paciente de 36 anos de idade e um caso de síndrome de Parkes-Weber em uma paciente de 21 anos. A literatura foi revisada com o objetivo de discutir as possíveis causas e consequências dessa doença e sua associação à hipertensão venosa e angiodisplasia. O presente trabalho também levanta discussão a respeito das diferenças sintomatológicas de ambas as síndromes e seus respectivos tratamentos.

## INTRODUCTION

According to the classification published by the International Society for the Study of Vascular Anomalies (ISSVA), Klippel-Trenaunay syndrome (KTS) is defined as capillary, venous, and lymphatic malformations associated with limb overgrowth, while Parkes-Weber syndrome (PWS) is characterized by the same triad of malformations combined with arteriovenous fistula.^1^ KTS and PWS are hardly ever seen in routine clinical practice, and they are commonly underdiagnosed conditions, furthermore, both syndromes have similar symptoms and are often confused during diagnosis.^2^

Both KTS and PWS can be associated with a variety of malformations and other symptoms, such as: hemimegalencephaly, genitourinary manifestations, developmental delays, polydactyly, macrodactyly, syndactyly, and seizures.^2^^-^ 7 The incidence of KTS appears to be 1:100.000 live births.^3^ Both syndromes mostly affect the lower limbs, although the upper limbs can be affected in rare cases.^3^^,^ 7

We present the cases of a 36-year-old male with KTS and a 21-year-old female with PWS. Both patients signed informed consent forms and this study conforms to the World Medical Association’s Helsinki Declaration.

## CASE REPORTS

### Case 1

**Figure 1:**
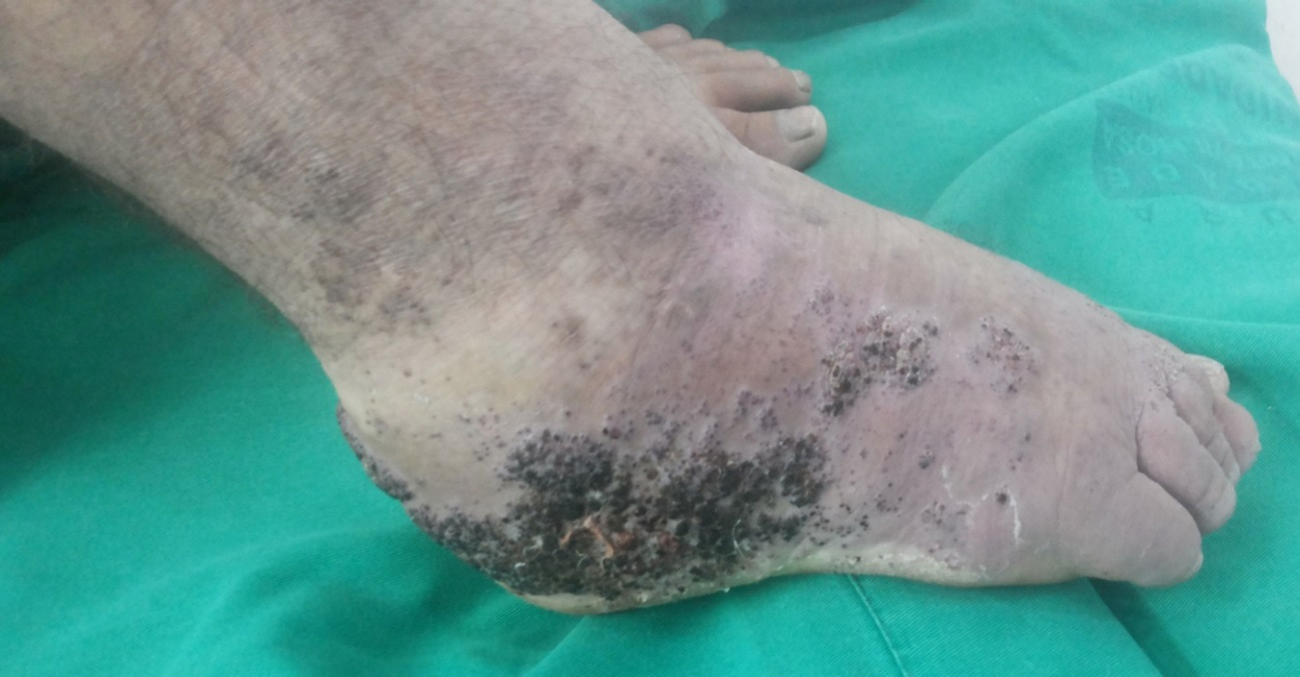
Verrucous nevi, lymphedema, venous angiodysplasia and angiokeratoma can be seen on the right lower foot.

A 36-year-old male sought our Angiology service in order to treat a venous ulcer on the right lower limb. Physical examination showed plain angioma on the right side of the body, more evident in the right lower limb, which had a verrucous nevi and angiokeratomas on the lateral surface of the foot (Figure 1).

We performed a color Doppler ultrasound examination of the right lower limb, which showed varicose veins and no signs of arteriovenous fistula, confirming the clinical diagnosis of KTS. We treated him with phlebotonics and drugs with lymphokinetic properties and conducted biannual follow-up while his ulcer was treated.

### Case 2

**Figure 2:**
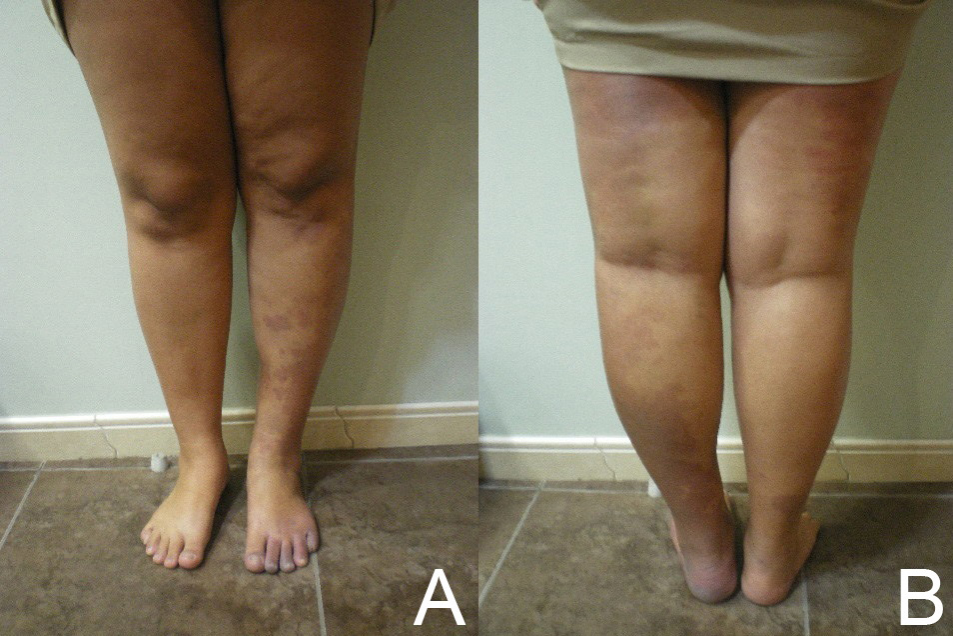
Disproportion between the second patient’s lower limbs. (A) anterior view; (B) posterior view.

A 21-year-old woman with a previous history of partial saphenectomy sought our Angiology service for treatment for a venous ulcer in the distal third of her left leg. She complained of joint pain and claudication. The ulcer had appeared one year after surgery. During the physical examination we observed angiomas on her left leg and disproportion between limbs (Figure 2).

**Figure 3:**
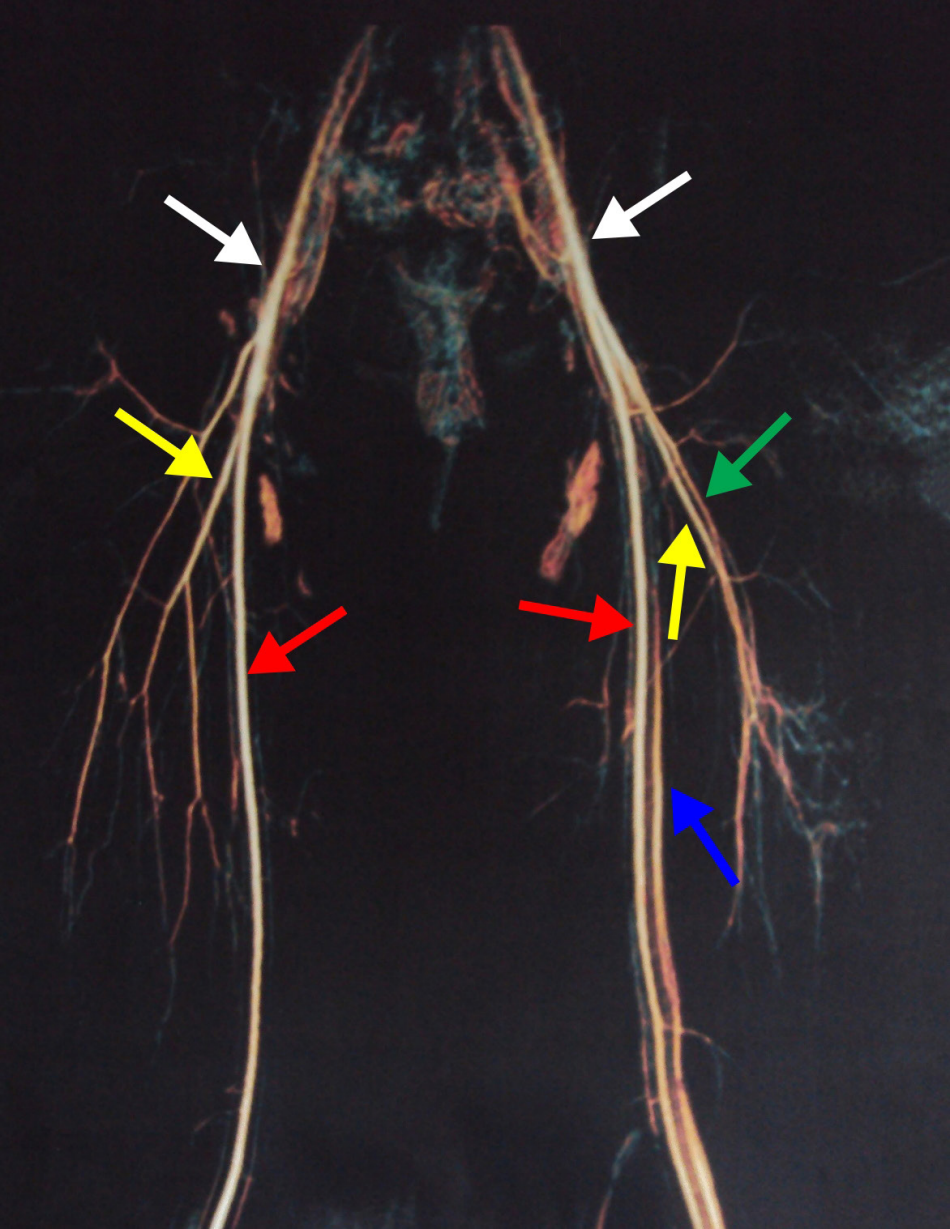
Magnetic resonance angiography of the second patient’s thighs. The examination shows an early venous flow on the right side, indicating an arteriovenous fistula. White arrows, common femoral artery; red arrows, superficial femoral artery; yellow arrows, deep femoral artery; blue arrow, left superficial femoral vein; green arrow, left deep femoral vein.

**Figure 4:**
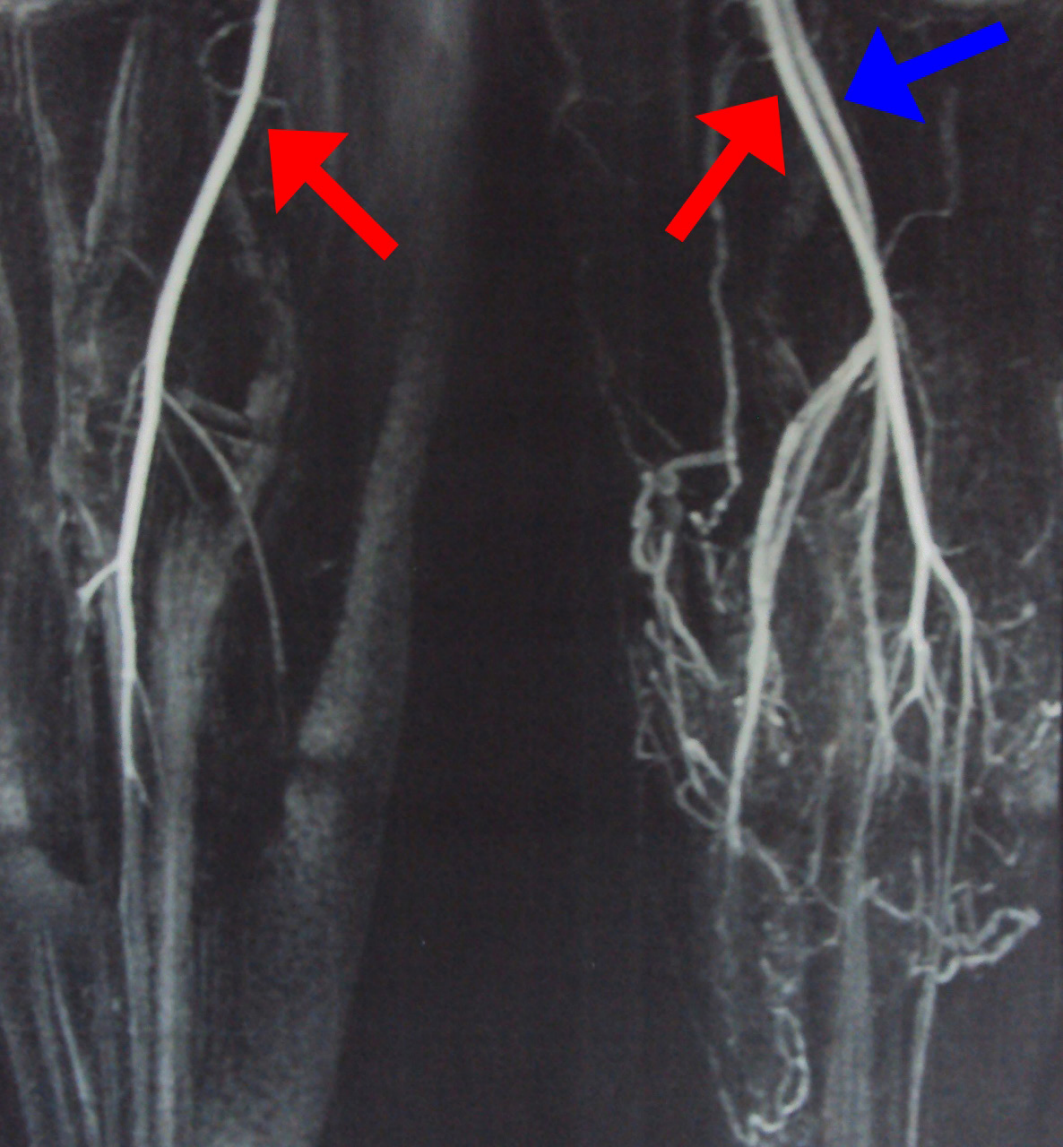
Magnetic resonance angiography of the second patient’s legs. Early venous filling during arterial phase of the exam, which indicates an arteriovenous fistula. Red arrows, popliteal artery; blue arrow, popliteal vein.

Arterial and venous color Doppler ultrasound and magnetic resonance angiography were used to confirm the initial KTS diagnosis. Doppler ultrasound and magnetic resonance angiography both showed hypoplasia of superficial and deep veins of the left lower limb, and an arteriovenous fistula, thereby confirming the diagnosis of PWS (Figures 3 and 4).

We requested stomach reduction surgery due to the patient’s elevated BMI and her joint and vascular symptoms improved. Her venous ulcer was treated successfully with compressive therapy.

## DISCUSSION

Both KTS and PWS are usually underdiagnosed conditions, furthermore, both syndromes have similar symptoms and are often confused during diagnosis.^2^

KTS and PWS occasionally cause genitourinary and gastrointestinal manifestations (both are significant sources of increased morbidity and mortality).^3^^,^ 8 ^,^ 9 KTS is usually associated with hemorrhages, hemimegalencephaly, genitourinary manifestations, developmental delays, polydactyly, macrodactyly, syndactyly, seizures thrombophlebitis, osteomyelitis, coagulopathy, pulmonary embolism, hemothorax, heart failure, hydronephrosis, predisposition to fractures, and in rare cases, ophthalmic alterations, such as conjunctival telangiectasia, orbital varix, strabismus, oculosympathetic palsy, Marcus-Gunn pupil, iris coloboma and heterochromia, cataracts, persistent fetal vasculature, and chiasmal and retinal varicosities.^3^^-^ 6 ^,^ 9 ^-^ 11

Patients with abnormal lymphatic drainage are at higher risk of contracting cellulitis and bacteremia. There are cases in which patients with KTS develop sciatic nerve hypertrophy causing pain,^12^ and even cases with congenital heart defects.^13^

Jacob et al.^14^ performed a study of 252 patients with KTS, and they concluded that port-wine hemangioma stains were found in 98%, varicose veins or venous malformations in 72%, and limb hypertrophy in 67%.

KTS increases the risk of thrombophlebitis and so oral contraceptives are contraindicated in female patients. Regular clinical and radiographic monitoring is crucial in order to assess progression or regression of the disease.^3^

On the other hand, PWS is associated with pulmonary manifestations such as thromboembolic phenomena, pulmonary venous varicosities, and pulmonary lymphatic obstruction,^7^ and there are also reports in the literature of rare cases with hydronephrosis and Kasabach-Merritt Coagulopathy.^8^ High output cardiac failure in PWS is usually secondary to anemia and large arteriovenous malformation, and can be fatal in pediatric patients.^7^ Varicose veins can affect both the deep and superficial venous systems and can predispose the affected limb to development of thrombophlebitis or venous ulcers.^7^

Pathophysiology has not been well established for either disease and their mechanisms of pathogenesis are also unclear, although there are theories described in the literature, such as: (1) congenital obstruction of the deep veins pertaining to the involved limb - usually the popliteal vein and associated varicose veins that drain directly to the internal iliac vein, causing circulatory overload;^9^ (2) mesodermal anomalies, which would justify poor formation of vascular and soft tissues during the fetal period;^15^ (3) mutations of genes that determine growth and cellular differentiation, combined with defects of the 5q chromosome (CMC1 locus), which is vital to angiogenesis.^5^^,^ 11 ^,^ 14 Newer studies have shown that KTS seems to be related to a mutation of the PIK3CA gene and that PWS is caused by mutations of the RASA1 gene, both genes that are responsible for mediating cellular growth, differentiation and proliferation (through the tyrosine kinase pathway).^4^^,^ 11 Moreover, it has been described that the pathways of mechanisms such as insulin-like growth factor, vascular endothelial growth factor, and fibroblast growth factor suffer malformation and overgrowth during embryogenesis.^10^

Both syndromes can be diagnosed by clinical examination, although supplementary exams are useful to confirm diagnosis and are essential to evaluate the stage of the disease.^2^ Radiological studies, such as ultrasound (with or without Doppler), computerized tomography (CT scan), magnetic resonance imaging (MRI) and vascular studies (arteriography and venography), can be valuable methods for differentiating these syndromes.^2^^,^ 6 ^,^ 14

Differential diagnosis for KTS and PWS should consider Proteus syndrome, Maffucci syndrome, neurofibromatosis type I, Sturge-Weber syndrome, and Beckwith-Wiedemann syndrome.^2^^,^ 3 ^,^ 5 ^,^ 7 ^,^ 9

Many different types of treatment have been used to manage KTS, including laser treatment;^9^ radiotherapy, cryotherapy, and sclerotherapy can produce damaging scars from an esthetic point of view. Surgery should be reserved for complicated cases in which the KTS is excessively symptomatic.^9^

Conventional sclerotherapy with liquid sclerosants can be used as a palliative treatment for KTS and PWS, as they produce many vascular abnormalities in the lower limbs. It offers good outcomes in patients with small malformations, and this technique is indicated as preoperative support in order to reduce the size of lesions before surgery or as a postoperative therapy.^16^

According to Yamaki et al.,^17^ foam sclerotherapy is more effective than liquid sclerotherapy for treatment of symptomatic venous malformations.

Microfoam sclerotherapy is a promising treatment for venous malformations associated with KTS and PWS because this simple procedure is capable of reducing or eliminating clinical symptoms even after the initial sessions, and since sclerotherapy is a minimally invasive procedure, it does not involve the risk of severe adverse reactions.^17^^-^ 19

Prescription of elastic compression stockings is useful in cases of chronic venous insufficiency (CVI) and lymphedema, with the purpose of preventing recurrent cellulitis and deep vein thrombosis, furthermore, it provides a source of protection during traumatic events, although it seems this course of management can cause problems for growing infants and small children.^9^^,^ 20

Patients with extensive lymphatic obstruction may have emotional and psychological traumas caused by limb deformity.^9^ Furthermore, vascular malformations do not regress, on the contrary, they become more prominent with time.^21^ Treatment of the capillary malformations with laser therapy could provide an improved cosmetic appearance.^21^

The patients described herein went through diet and/or stomach reduction surgery, and since weight loss seemed to improve their symptoms greatly we would also include a change in dietary habits as a complementary treatment in both diseases.

Chagas et al.^20^ emphasize the congenital and gestational character of these angiodysplasias. Furthermore, they also emphasize the osteodysplasia commonly associated with vascular alterations in a great number of cases and therefore classify these eponymous diseases using the generic term “angiodysplasias”, in order to avoid confusion.

There is no uniform clinical presentation of angiodysplasia cases, since they can have greater or lower numbers of associated symptoms and properly taken patient history and thorough physical examination are needed to elucidate the diagnosis of angiodysplasia.^20^

We conclude that KTS and PWS, are underdiagnosed and dangerous disorders that merit further study, since their symptoms can be devastating and they have many complications. Furthermore, both diseases can deeply affect patients’ psychological wellbeing, as seen in one of our reports, negatively impacting their quality of life.
